# Frequency of Workplace Burnout Among Postgraduate Trainees in a Teaching Hospital in Mirpur

**DOI:** 10.7759/cureus.4016

**Published:** 2019-02-05

**Authors:** Abid Naeem, Altaf A Shaikh, Subtain Ul Hassan Abid, Huzaifa Abid, Amber Tahir

**Affiliations:** 1 Internal Medicine, Mohi-Ud-Din Islamic Medical College, Mirpur, PAK; 2 Internal Medicine, Ghulam Mohammad Mahar Medical College and Hospital, Sukkur, PAK; 3 Internal Medicine, Combined Military Hospital, Jhelum, PAK; 4 Family Medicine, Chandka Medical College Hospital, Larkana, USA; 5 Internal Medicine, Dow University of Health Sciences, Karachi, PAK

**Keywords:** american welfare association burnout questionnaire, azad kashmir, burnout syndrome, physician burnout, postgraduate trainees, reasons of burnout

## Abstract

Background: Work-related stress and burnout among medical practitioners has been a frequently studied phenomenon. It reduces work efficiency and productivity and also has negative impacts on patient care. This study assessed the extent of work-related stress and burnout and the reasons behind this burnout among the Internal Medicine and Pulmonology residents of Mirpur, Azad Kashmir.

Materials and methods: This cross-sectional, observational study was conducted among the postgraduate trainees of Internal Medicine and Pulmonology in Mohi-uddin Islamic Medical College and Hospital, Mirpur, Azad Kahsmir. Out of the 70 trainees, 64 completed the study (response rate: 85.3%). The trainees recorded their sociodemographic profile including age, gender, marital status, name of the department, and year of training. Work-related stress and burnout were assessed using a 28-question-based Burnout Questionnaire adapted from the American Welfare Association. Reasons of burnout among the postgraduate trainees were recorded. Data were analyzed using SPSS v. 21.

Results: There were 45 men (70.3%) and 19 women (29.6%). The mean age of the trainees was 29.25 ± 2.87 years. No stress and professional burnout was reported in 20.3% residents; 29.6% residents had stress but no professional burnout; 23.4% residents had fair chances of burnout; 14% residents had early burnout; and 12.5% residents had advanced burnout. Higher grades of burnout are more common among male residents, those who are married, and those in early years of postgraduate training. Common reasons of work-related burnout were reported to be long work hours (68.75%), decreased job satisfaction (54.7%), and lack of workplace facilities (45.3%).

Conclusion: Most of the residents in Mirpur have work-related stress and are at the verge of burning out. Large-scale studies, assessing more correlates, must be conducted in this region to give a better understanding of this phenomenon and help formulate plans to prevent and manage work-related stress and burnout among the postgraduate trainees.

## Introduction

Burnout from chronic stress among medical students, postgraduate trainees, and consultant physicians and surgeons is not a new observation, but has been previously underestimated. Burnout is a result of chronic exposure to interpersonal as well as emotional job stressors. According to Maslach, burnout is essentially relevant to medical practitioners because of the significant disparity between available resources and work demands which results in exhaustion and fatigue [1].

Burnout in medical practitioners implicates negative effects on job performance. It results in disconnection with work, disappointment, lowered self-esteem, and the sense of not advancing towards achievement of life goals. It reduces work efficiency and job satisfaction and increases the ratio of absenteeism and job turnover [2]. Burnout also causes adverse impacts on physical health including chronic fatigue, body pains, headache, decreased sleep quality, and respiratory and gastrointestinal disturbances [3].

IIn a recent systematic review of 182 studies, the overall prevalence of physician burnout was reported to be 67% (range: 0%-80.5%). As far as subcomponents were concerned, emotional exhaustion was reported in 72% studies, depersonalization in 68.1% studies, and low personal accomplishment was reported in 63.2% studies [4]. In a survey conducted in Pakistan among resident trainees, it was concluded that 74.4% residents reported high burnout on at least one subscale of Maslach Burnout Inventory. Highest burnout was reported on the subscale of emotional exhaustion (59.8%). They reported long working hours, workload, inter-personal relations, and lack of autonomy as reasons for burnout [5].

Although we could not find any study which evaluated the levels of burnout among Kashmiri physicians, a study reported lack of financial rewards, inflexible working hours, lack of autonomy, personal issues, and bureaucratic system of management to reduce work productivity of Kashmiri public sector employees [6]. Furthermore, in a study conducted with Indian-Kashmiri critical care doctors, 43.75% of them were reported to have moderate to severe stress [7]. Hence, this is the first of its kind study from Mirpur, the capital and the largest city of Azad Kashmir. This study assesses the extent of burnout among the postgraduate trainees and the reasons behind this burnout in this region.

## Materials and methods

This cross-sectional, observational study was conducted from September till December 2018 among postgraduate trainees of Internal Medicine and Pulmonology in Mohi-uddin Islamic Medical College and Hospital, Mirpur, Azad Kahsmir. There are a total of 70 trainees inducted in both these departments combined. All were invited to participate in the study, however, 66 trainees agreed to participate. Two responses were omitted due to incomplete questionnaires; hence, 64 trainees completed the study which makes a response rate of 85.3%. A 28-question-based Burnout Questionnaire was adapted from the American Welfare Association [8]. As done by Bedre et al. [9], the scores were stratified to assess the severity of burnout among postgraduate trainees (Table 1).

**Table 1:** Stratification of burnout score of the American Welfare Association Burnout Scale.

Grades of burnout	Scores	Inferences
I	28-38	No stress and professional burnout
II	38-50	Stress but no professional burnout
III	51-70	Fair chances of burnout
IV	71-90	Early burnout
V	90+	Advanced burnout

The trainees also recorded their sociodemographic profile including age, gender, marital status, name of the department, and year of training. Workplace-related reasons of burnout among the postgraduate trainees were also recorded on open-ended questions. Data were analyzed using SPSS v. 21. 

## Results

Out of the 64 study participants, there were 45 men (70.3%) and 19 women (29.6%). The mean age of the trainees was 29.25 ± 2.87 years. There were 25 married trainees (39%) and 39 unmarried trainees (60.9%). There were 50 trainees (78.1%) from the department of Internal Medicine and 14 (21.9%) from the department of Pulmonology. There were 42 trainees (65.6%) from first and second year and 22 trainees (34.4%) from third and fourth year of postgraduate training.

No stress and professional burnout (Grade I) was reported in 20.3% residents; 29.6% residents had stress but no professional burnout (Grade II); 23.4% residents had fair chances of burnout (Grade III); 14% residents had early burnout (Grade IV); and 12.5% residents had advanced burnout (Grade V). The extent of burnout stratified according to the sociodemographic profile is given in Table 2.

**Table 2:** Degree of burnout in correlation with sociodemographic profile (N = 64).

Degree of burnout	Gender n (%)		Marital status n (%)		Department n (%)		Year of training n (%)	
	Male	Female	Married	Unmarried	Internal medicine	Pulmonology	1^st^ and 2^nd^ year	3^rd^ and 4^th^ year
Grade I (n = 13/64; 20.3%)	9/13 (69.2%)	4/13 (30.8%)	2/13 (15.4%)	11/13 (84.6%)	8/13 (61.5%)	5/13 (38.5%)	9/13 (69.2%)	4/13 (30.8%)
Grade II (n = 19/64; 29.6%)	15/19 (78.9%)	4/19 (21.1%)	5/19 (26.3%)	14/19 (73.7%)	13/19 (68.4%)	6/19 (31.6%)	13/19 (68.4%)	6/19 (31.6%)
Grade III (n = 15/64; 23.4%)	11/15 (73.3%)	4/15 (26.7%)	6/15 (40%)	9/15 (60%)	12/15 (80%)	3/15 (20%)	10/15 (66.7%)	5/15 (33.3%)
Grade IV (n = 09/64; 14%)	5/9 (55.5%)	4/9 (44.5%)	6/9 (66.7%)	3/9 (33.3%)	9/9 (100%)	0/9 (0%)	4/9 (44.5%)	5/9 (55.5%)
Grade V (n = 08/64; 12.5%)	5 (62.5%)	3 (37.5%)	6 (75%)	2 (25%)	8 (100%)	0 (0%)	6 (75%)	2 (25%)

The trainees recorded their reasons of burnout in open-ended questions. The responses were stratified into categories as presented in Table 3.

**Table 3:** Workplace-related reasons of burnout among the participants (N = 64).

Workplace-related reasons of burnout	Frequency n (%)
Stretched working hours	44 (68.75%)
Decreased job satisfaction	35 (54.7%)
Unavailability of workplace facilities	29 (45.3%)
Lack of career opportunities	19 (29.6%)
Low salaries as compared to the workload	12 (18.75%)
Highly competitive work environment	09 (14%)

## Discussion

This study evaluated the extent of burnout among the postgraduate trainees of two main specialties—internal medicine and pulmonology. Grade II—stress but no professional burnout—was the most common (29.6%). Grade V—advanced burnout—was the least common (12.5%). Higher grades of burnout are more common among male residents, those who are married, and those in early years of postgraduate training. The common factors associated with resident stress and burnout were long, continuous work hours, dissatisfaction with job, and lack of workplace facilities.

Although burnout had been frequently reported among various healthcare practitioners, data were missing from this part of the world. This is the first of its kind study to evaluate resident physician burnout from Mirpur, Azad Kashmir. This study has its limitations too. It did not utilize the most common and reliable burnout inventory—the Maslach Burnout Inventory—due to lack of funds. It also did not include residents from other subspecialties of medicine and residents of surgery.

Stretched work hours have been a global issue for trainee doctors. It has long been regarded as the culprit to reduced resident well-being [10-11] along with compromised patient care [11-12]. Nonetheless, studies failed to depict any significant betterment in resident burnout levels even after implementing controlled working hours [13-14]. In a comparative study which reported 56.3% resident trainees to be “under stress,” a positive correlation was established between mean working hours and job stress as well as job satisfaction [15]. Work-related burnout has been reported to be among 41% of medical residents in India which was more for third year residents (52.58%) and female residents (37.07%) [16]. On the contrary, our study reported higher grades of burnout among residents of early years of training.

Although this study did not report any dramatically high frequency of burnout among postgraduate residents of Mirpur, it did signify that many of the residents are stressed and at the verge of burning out. Hence, this first of its kind study from this region holds crucial value. We recommend further studies from this region, especially using Maslach Burnout Inventory, and interventional studies that can help lower overall stress level among these residents.

## Conclusions

Most of the residents in Mirpur have work-related stress and are at the verge of burning out. Higher grades of burnout are more common among male residents, those who are married, and those in early years of postgraduate training. Large-scale studies, assessing more correlates, and comparing residents of various medical specialties as well as comparing stress and burnout in medical trainees with that in the surgical trainees need to be conducted. These will provide with better understanding of this phenomenon and will help devise measures to prevent and manage work-related stress and burnout among postgraduate trainees.
